# Preliminary research on LncRNA ATP2B2-IT2 in neovascularization of diabetic retinopathy

**DOI:** 10.1186/s12886-024-03523-5

**Published:** 2024-06-21

**Authors:** Yuan Yuan, Anming Zhu, Lan Zeng, Xiaocong Wang, Ying Zhang, Xiaofeng Long, Jie Wu, Meng Ye, Junhao He, Wei Tan

**Affiliations:** 1 Department of Ophthalmology, Zunyi First People’s Hospital, The Third Affiliated Hospital of Zunyi Medical University, Zunyi, China; 2 Zunyi Medical University, Zunyi, China; 3 Department of Ophthalmology, The Third Hospital of Mianyang, Mianyang, China; 4 Scientific Research Center, The First People’s Hospital of Zunyi (The Third Affiliated Hospital of Zunyi Medical University), Zunyi, 563000 China; 5 Yunyang county people’s hospital, Yunyang, 404500 China

**Keywords:** Diabetic retinopathy, LncRNA ATP2B2-IT2, Proliferation, Migration, Neovascularization

## Abstract

**Objective:**

Diabetic retinopathy (DR) is a common complication of diabetes, and recent findings have shown that long noncoding RNAs (lncRNAs) may be involved in its pathogenesis. Through bioinformatics analysis, we found that lncRNA ATP2B2-IT2 may be involved in this process. This study primarily investigated the expression of the lncRNA ATP2B2-IT2 in human retinal microvascular endothelial cells (HRMECs) under high-glucose conditions and its effects on HRMEC proliferation, migration, and neovascularization.

**Methods:**

We used RT‒PCR to assess the expression levels of lncRNA ATP2B2-IT2 and vascular endothelial growth factor (VEGF) in HRMECs under normal glucose (5.5 mmol/L) and high glucose (30 mmol/L) conditions. HRMECs were subsequently divided into four groups: the normal glucose (NG), high glucose (HG), high glucose with lncRNA ATP2B2-IT2 silencing (HG + si-lncRNA ATP2B2-IT2), and high glucose with silencing control (HG + si-NC) groups. The expression levels of the lncRNA ATP2B2-IT2 and VEGF in each group were determined using RT‒PCR. Thereafter, cell proliferation, migration, and neovascularization were assessed using CCK-8, Transwell, and tube formation assays, respectively.

**Results:**

RT‒PCR revealed that the expression levels of the lncRNA ATP2B2-IT2 and VEGF were greater in the HG group than in the NG group (*P* < 0.05). After silencing of the lncRNA ATP2B2-IT2, the expression of VEGF decreased significantly (*P* < 0.05). Subsequent CCK-8, Transwell, and tube formation assays demonstrated that compared to those in the NG group, the HRMECs in the HG group exhibited significantly increased proliferation, migration, and neovascularization (*P* < 0.05). However, after silencing of the lncRNA ATP2B2-IT2, the proliferation, migration, and neovascularization of HRMECs were significantly decreased in the HG + si-lncRNA ATP2B2-IT2 group compared to those in the HG group (*P* < 0.05).

**Conclusion:**

LncRNA ATP2B2-IT2 may promote the proliferation, migration and neovascularization of HRMECs under high-glucose conditions.

**Supplementary Information:**

The online version contains supplementary material available at 10.1186/s12886-024-03523-5.

## Introduction

Diabetes is a worldwide health problem [1]. The damage caused by diabetes to all body organs mainly manifests as macrovascular and microvascular complications, including diabetic retinopathy [2–5]. Diabetic retinopathy (DR) is a common retinal microvascular disease and is the primary cause of vision loss in diabetic patients [6, 7]. Without active treatment and with progression to an advanced stage, DR can seriously affect the vision and self-care ability of diabetic patients. Clinically, DR can be classified into NPDR and PDR [8]. Nonproliferative DR refers to early-stage DR characterized by microhemorrhages, microaneurysms, and increased retinal permeability [9]. Neovascularization indicates the progression of DR from the early to the advanced and most severe stage when proliferative DR (PDR) occurs [10].

Existing treatments for DR target neovascularization in advanced PDR and include retinal lasers, anti-VEGF treatment, and surgery [11, 12]. Prevention is the major measure for early-stage DR, and there are no aggressive or effective treatments available to stop the progression of DR. Moreover, the specific process of neovascularization in PDR is unclear and remains to be determined. In a preliminary study, vitreous humor (VH) samples from patients diagnosed with PDR and non-PDR patients were utilized as the experimental group. The expression levels of lncRNAs were assessed through gene chip analysis, revealing differential expression of lncRNAs between the two groups. Subsequently, further screening was conducted on the differentially expressed lncRNA data, focusing on genes with a log fold change (logFC) greater than 2 and a corrected *P* value less than 0.05. The lncRNA ATP2B2-IT2 was identified as one of the genes with significantly differential expression; thus, we focused on this molecule for subsequent investigations. Therefore, we focused on the lncRNA ATP2B2-IT2 to study the neovascularization process in PDR, thus providing insight for the early treatment of DR.

## Materials and methods

### Materials and reagents

In this study, human retinal microvascular endothelial cells (HRMECs) were purchased from Beijing Beina Chuanglian Biotechnology Research Institute. The sequences used to silence the lncRNA ATP2B2-IT2 were designed and synthesized by Shanghai GenePharma. Three primers, namely, primers for lncRNA ATP2B2-IT2, VEGF, and β-actin, were designed and synthesized by Invitrogen. The pcDNA-si ATP2B2-IT2 and pcDNA-3.1 vectors were obtained from Shanghai GenePharma. Three silencing sequences for the lncRNA ATP2B2-IT2 were designed and synthesized by Shanghai GenePharma (as shown in Table 1).

**Table 1:** Primer sequences for the lncRNA ATP2B2-IT2

Name	Sequence
LncRNA ATP2B2-IT2#1	S:5’-GGUUCUCACUAAGCAUCAATT-3’
	AS: 5’-UUGAUGCUUAGUGAGAACCTT-3’
LncRNA ATP2B2-IT2#2	S: 5’-CCAAGAGAGACAUUCACAUTT-3’
	AS: 5’-AUGUGAAUGUCUCUCUUGGTT-3’
LncRNA ATP2B2-IT2#3	S: 5’-CCAAACUCCAGUUGUUUCUTT-3’
	AS: 5’-AGAAACAACUGGAGUUUGGTT-3’

### Main reagents

ECM medium (ScienCell, USA), 2x SYBR Green PCR mix and 4% paraformaldehyde (Soleibaol, Beijing), CCK-8 and crystal violet stain (Biyuntian Biotechnology Company, Shanghai), matrix glue (Corning, United States), and Lip2000 Transfection Reagent (Biosharp, China) were used.

### Cell culture

HRMECs were cultured with ECM Endothelial Cell Medium in a cell culture incubator under a humid atmosphere with 5% CO_2_.

### Cell transfection and grouping

The cells were inoculated in a 6-well plate at 2 × 10^5^ cells per well and transfected after 24 h of incubation. HRMECs were subsequently divided into four groups, namely, the NG, HG, HG + si-lncRNA ATP2B2-IT2, and HG + si-NC groups. Lipofectamine^™^ 2000 was used to transfect the empty vectors pcDNA3.1-si ATP2B2-IT2 and pcDNA3.1 at a concentration of 50 nmol/L.

### RNA extraction and RT‒PCR

After the total RNA of the HRMECs in each group was extracted using TRIzol reagent (Thermo Scientific, USA), a NanoDrop LITE spectrophotometer (Thermo Scientific, USA) was used to measure the concentration of RNA in each group. The resulting RNA was reverse transcribed by the PrimeScriptTM RT Reagent Kit Reverse Transcription System (TaKaRa, Japan) and quantified by a CFX96 real-time quantitative PCR system (Bio-Rad, USA). The sequences of primers used were 5’-GATCCAAGAGATGCAGAGGCTAAGC-3’ (the forward primer for the lncRNA ATP2B2-IT2), 5’-TGTGGGAGAGGCAGGCTTCAGAG-3’ (the reverse primer for the lncRNA ATP2B2-IT2), 5’-CTTCGCTTACTCTCACCTGCTTCTG-3’ (the forward primer for VEGF), 5’-GC TGTCATGGGCTGCTTCTTCC-3’ (the reverse primer for VEGF), 5’-CCTTCCTGGGCATGGGAGTC-3’ (the forward primer for β-actin), and 5’-TGATCTTCATTGTGCTGGGGTG-3’ (the reverse primer for β-actin). The relative expression of the lncRNA ATP2B2-IT2 and VEGF was calculated using the formula 2^-△△Ct^.

### Analysis of proliferation

A CCK-8 assay was used to measure proliferation. HRMECs from four groups (NG, HG, HG + si-NC, and HG + si-lncRNA ATP2B2-IT2) were inoculated in a 96-well plate with 5 × 10^4^ cells per well. After 24 h of incubation, 110 ml of ECM medium-CCK-8 mixture was added to each well at a ratio of 10:1, the plates were incubated for 1 h, and the plates were subsequently placed in an i3x multifunctional enzyme labeling detector (Molecular Devices, USA) to determine the absorbance of the cells at 450 nm to obtain the OD. The experiment was repeated three times under the same conditions.

### Measurement of migration

Cell migration was measured by Transwell assays. After the HRMECs in the four groups (NG, HG, HG + si-NC, and HG + si-lncRNA ATP2B2-IT2) were digested with trypsin, the HRMECs were diluted to a density of 4 × 10^5^ cells/ml in complete NG/HG ECM (5% FBS) and inoculated in the chambers of a 24-well Transwell plate. Next, 500 µL of complete NG/HG ECM (20% FBS) was added to the cell culture incubator (37 °C, 5% CO_2_, 95% humidity) in the lower chambers to culture 48 chambers of HRMECs. The inner cells of the microporous membrane were removed with a moistened cotton swab and washed with PBS 3 times, while the outer cells of the microporous membrane of the chamber were fixed with 4% formaldehyde for 20 min and stained with 0.1% crystal violet for 30 min. After that, the microporous membrane was removed, placed on a slide, and prepared by placing a drop of neutral gum on the slide. After preparation, the cells were observed and photographed under a microscope, and the images were processed in ImageJ. The Transwell assay was repeated three times, and the average of the results was taken.

### Measurement of the tube-forming ability

A tube formation assay was used to measure the tube-forming ability of the cells. Then, 50 µL/well Matrigel matrix gel was added to a 96-well plate, which was placed in a cell incubator (37 °C, 5% CO_2_, 95% humidity) for 1 h for solidification. Next, NG, HG, HG + si-NC, and HG + si-lncRNA ATP2B2-IT2 cells were inoculated at a density of 4 × 10^5^ cells/ml in a 96-well plate. After 8 h of inoculation, the 96-well plate was removed and placed under a microscope for observation and imaging, and the images were processed in ImageJ. The experiment was repeated three times, and the experimental results were averaged.

### Statistical analyses

The above experiments were repeated three times in parallel. The measurement results are expressed as the mean ± standard deviation (X ± SD) if they were normally distributed. ImageJ 1.8.0 software was used to analyze the experimental data, and GraphPad Prism 8 software was used for statistical analysis and graphing of the experimental data. Independent samples t tests were used to analyze the variance between two groups, and one-way ANOVA was used to compare more than two groups. When the data did not satisfy the normal distribution, the rank sum test was used. *P* < 0.05 indicated statistical significance.

## Results

### Upregulation of lncRNA ATP2B2-IT2 expression and VEGF expression in HRMECs under high-glucose conditions

**Fig. 1:**
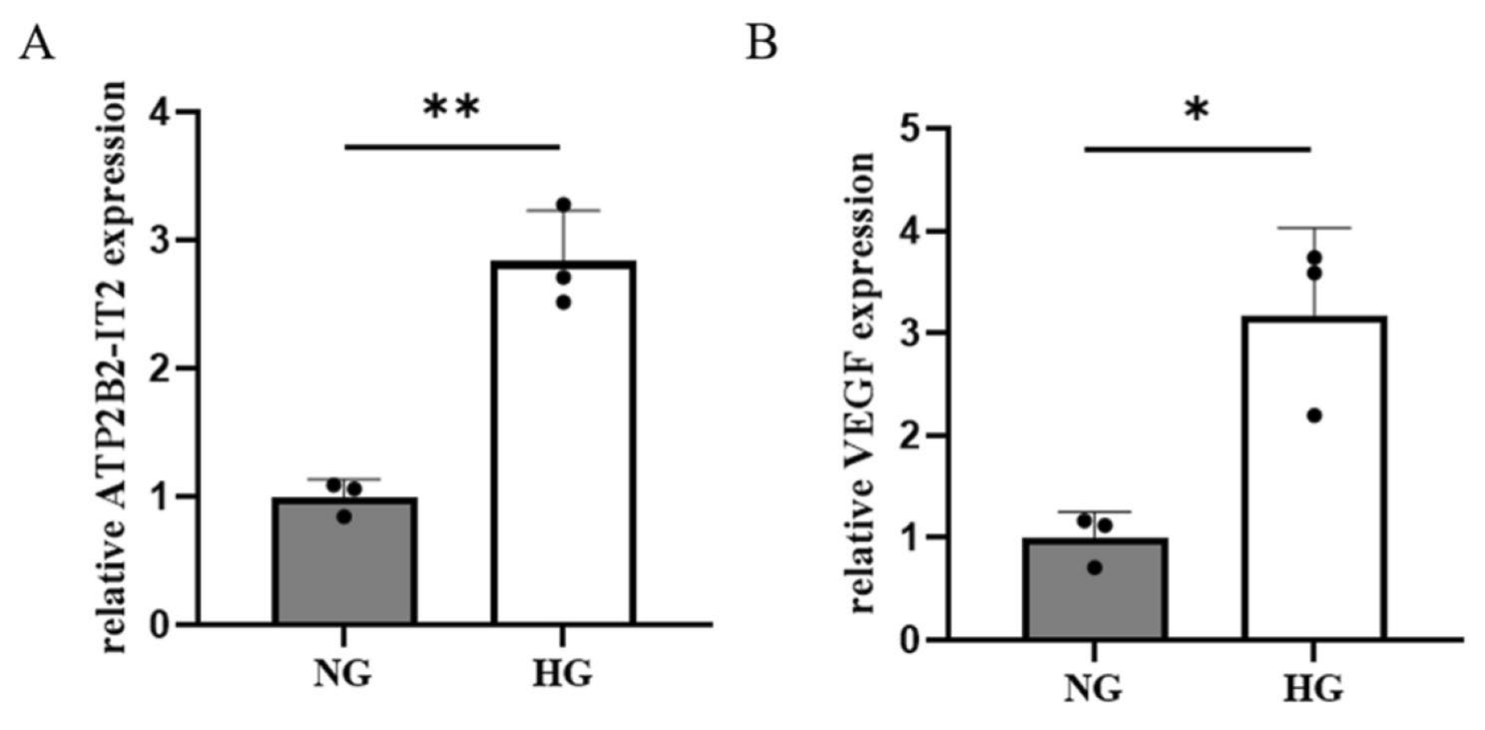
Expression of the lncRNA ATP2B2-IT2 and VEGF in HRMECs cultured in a high-glucose environment (*P < 0.05, **P < 0.01, ***P < 0.001); (**A**)The expression of lncRNA ATP2B2-IT2 in the HRMECs of individuals under NG and HG; (**B**) The expression of VEGF in the HRMECs of individuals under NG and HG

According to the RT‒PCR results, lncRNA ATP2B2-IT2 expression was significantly upregulated in the HG group compared with the NG group, and the difference between the two groups was significant (*P* < 0.05). VEGF expression was significantly upregulated in the HG group compared with the NG group, and the difference between the two groups was significant (*P* < 0.05), as shown in Fig. 1.

### Selection of the transfection reagent

**Fig. 2:**
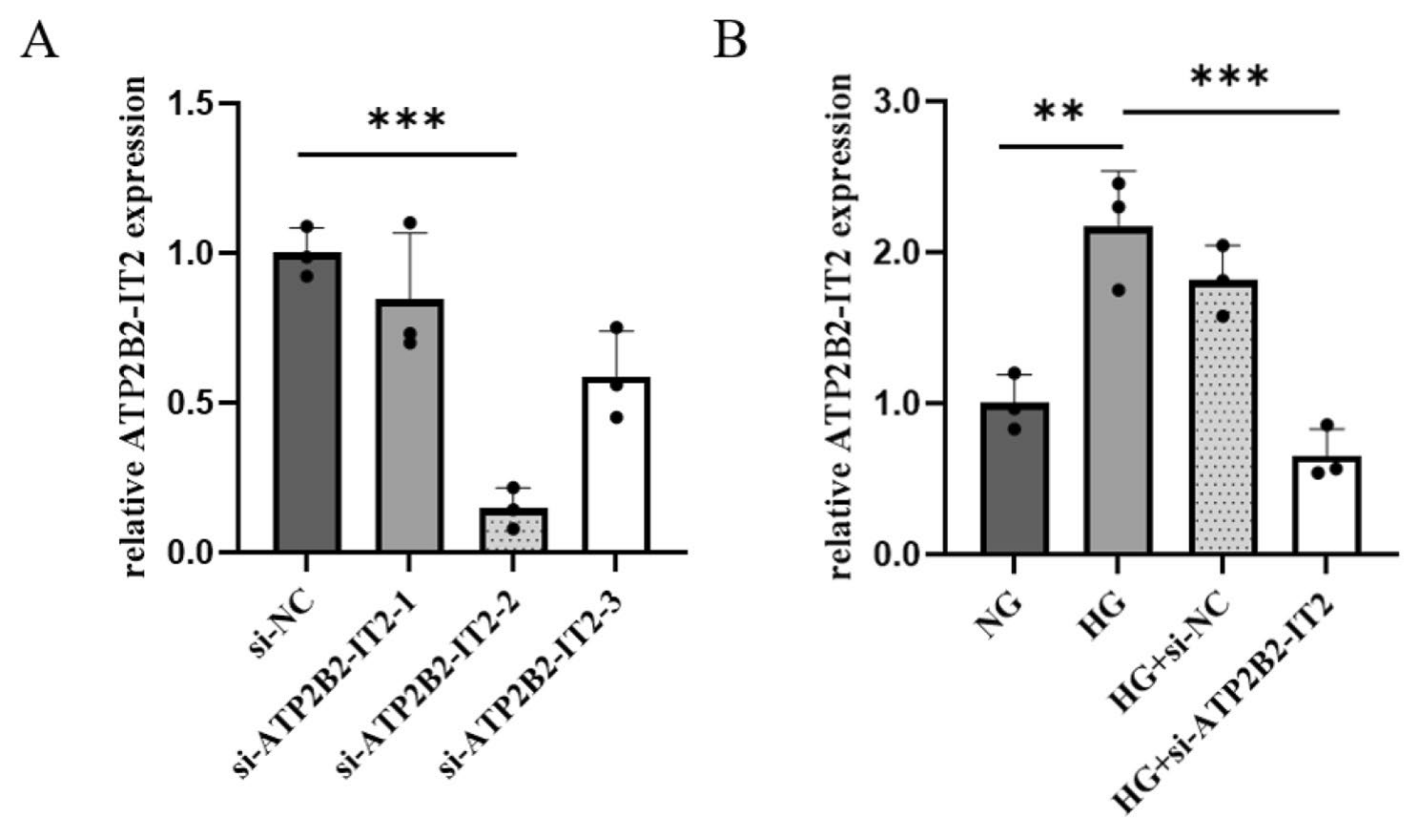
Expression of three lncRNA ATP2B2-IT2 silencing sequences in HRMECs cultured in a high-glucose environment (*P < 0.05, **P < 0.01, ***P < 0.001); (**A**) The expression status of three si-ATP2B2-IT2 under high HG. (**B**) The comparison of the expression status of si-ATP2B2-IT2 under HG with other groups

RNA was extracted from cells transfected with si-lncRNA ATP2B2-IT2, and RT‒PCR was performed to determine the expression of the lncRNA ATP2B2-IT2. The results showed that among the three siRNA sequences, Sequence 2 had the highest transfection efficiency, and its difference from the other two sequences was significant (*P* < 0.05); therefore, Sequence 2 was selected as the siRNA for the subsequent experiments, as shown in Fig. 2.

### Effect of si-lncRNA ATP2B2-IT2 on the proliferation of HRMECs under high-glucose conditions

**Fig. 3:**
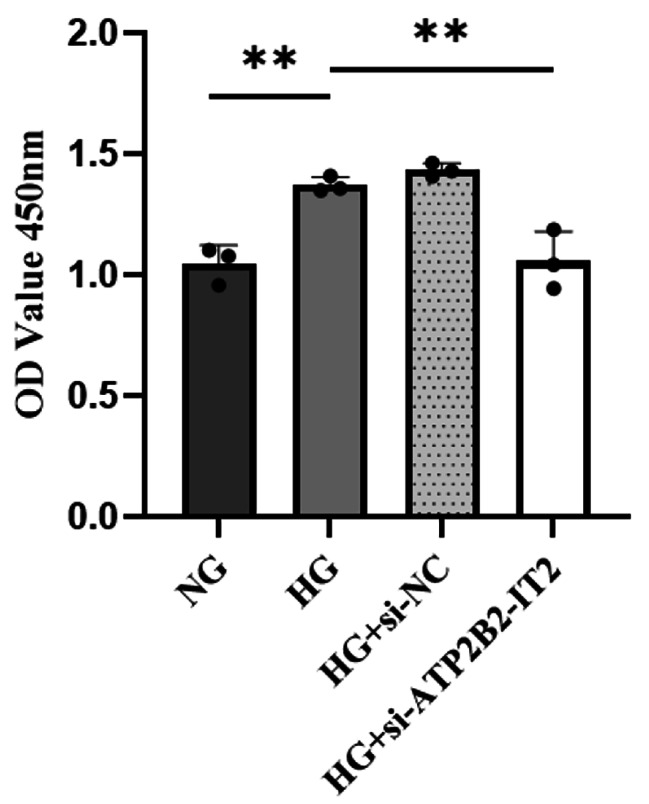
Effect of lncRNA ATP2B2-IT2 silencing on the proliferation of HRMECs induced by high glucose (*P < 0.05, **P < 0.01, ***P < 0.001)

The proliferation of HRMECs was measured by CCK-8 assays. According to the CCK-8 assay results, the proliferative activity of HRMECs in the HG group was greater than that in the NG group, and the difference between the two groups was significant (*P* < 0.05); the proliferative activity of HRMECs in the HG + si-lncRNA ATP2B2-IT2 group was significantly less than that in the HG group (*P* < 0.05), but the proliferative activity of HRMECs in the HG + si-lncRNA ATP2B2-IT2 group and the HG + si-NC group did not significantly differ (*P* > 0.05), as shown in Fig. 3.

### Effect of si-lncRNA ATP2B2-IT2 on the migration of HRMECs under high-glucose conditions

**Fig. 4:**
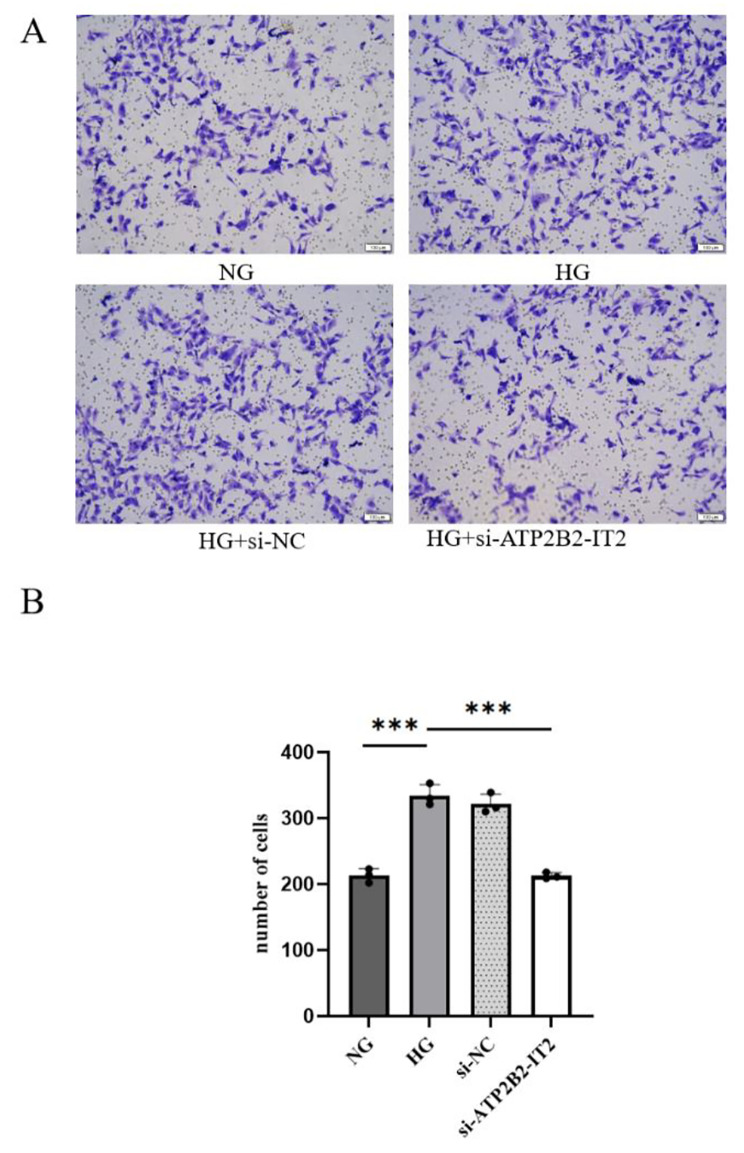
Effect of si-lncRNA ATP2B2-IT2 on the migration of HRMECs under high-glucose conditions; (**A**) Representative images of migrated cells in each group (scale = 100 μm); (**B**) Number of migrated cells in each group (*n* = 3; *P < 0.05, **P < 0.01, ***P < 0.001)

A Transwell assays were used to assess the migration of HRMECs. According to the results of the Transwell assay, the number of migrating HRMECs in the HG group was significantly greater than that in the NG group, and the difference between the two groups was significant (*P* < 0.05). The number of migrated HRMECs in the HG + si-lncRNA ATP2B2-IT2 group was significantly lower than that in the HG group (*P* < 0.05), and the number of migrated HRMECs in the HG + si-lncRNA ATP2B2-IT2 group and the HG + si-NC group did not significantly differ (*P* > 0.05), as shown in Fig. 4.

### Effect of si-lncRNA ATP2B2-IT2 on the tube formation of HRMECs under high-glucose conditions

**Fig. 5:**
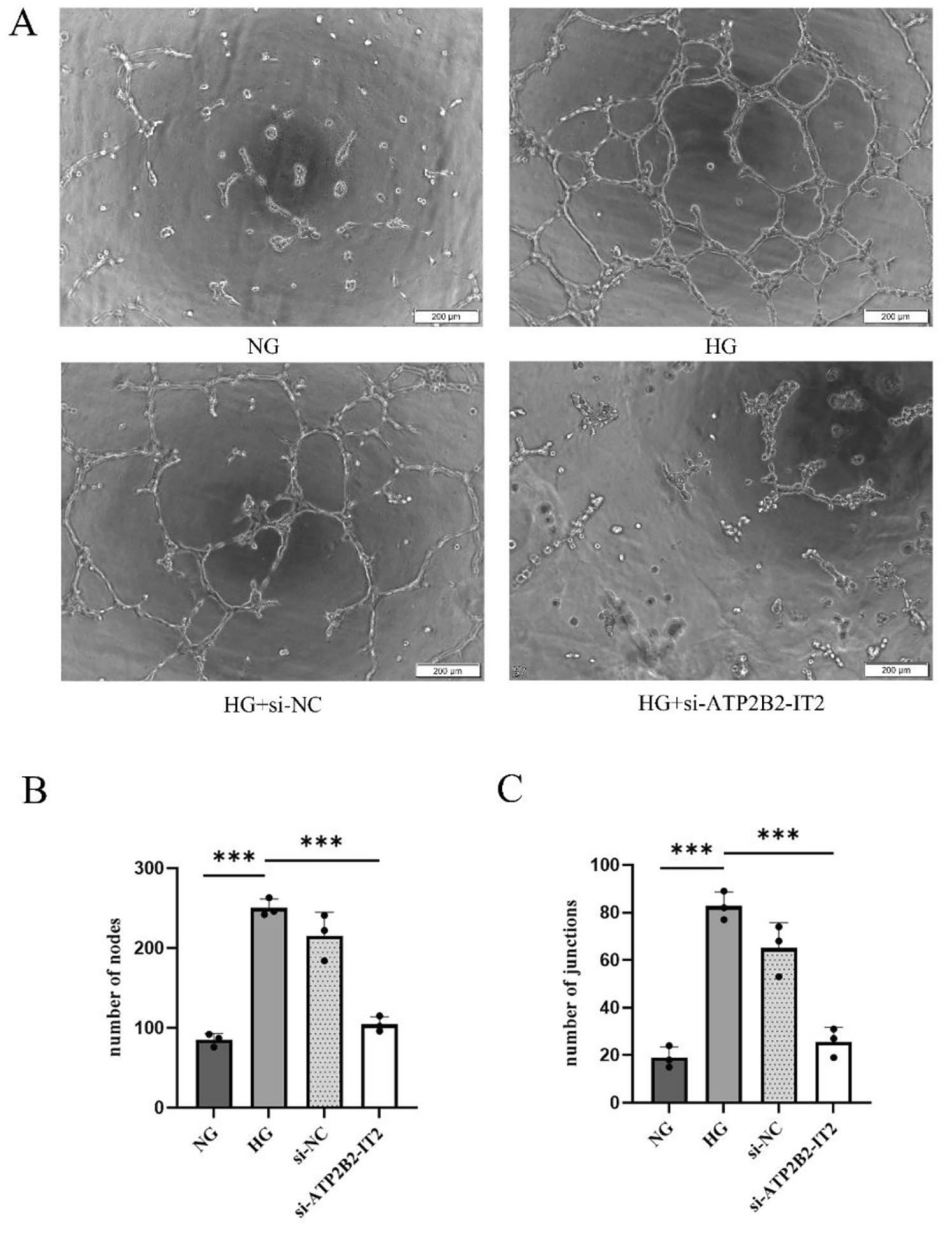
Effect of si-lncRNA ATP2B2-IT2 on the tube formation of HRMECs under high-glucose conditions (*n* = 3; *P < 0.05, **P < 0.01, ***P < 0.001); (**A**) Representative images of tube-forming nodes and branches in each group (scale = 200 μm); (**B**) Number of tube-forming nodes in each group; (**C**) Number of tube-forming branches in each group

A tube formation assay was used to measure the angiogenic ability of HRMECs. The assay results showed that the number of tubes formed by the HRMECs of the HG group was significantly greater than that of the NG group, and the difference between the two groups was significant (*P* < 0.05); the number of tubes formed by the HRMECs of the HG + si-lncRNA ATP2B2-IT2 group was significantly smaller than that in the HG group (*P* < 0.05), while there was no significant difference between the HG + si-lncRNA ATP2B2-IT2 group and the HG + si-NC group (*P* > 0.05), as shown in Fig. 5.

### Downregulation of VEGF expression in HRMECs under high-glucose conditions after silencing of the lncRNA ATP2B2-IT2

**Fig. 6:**
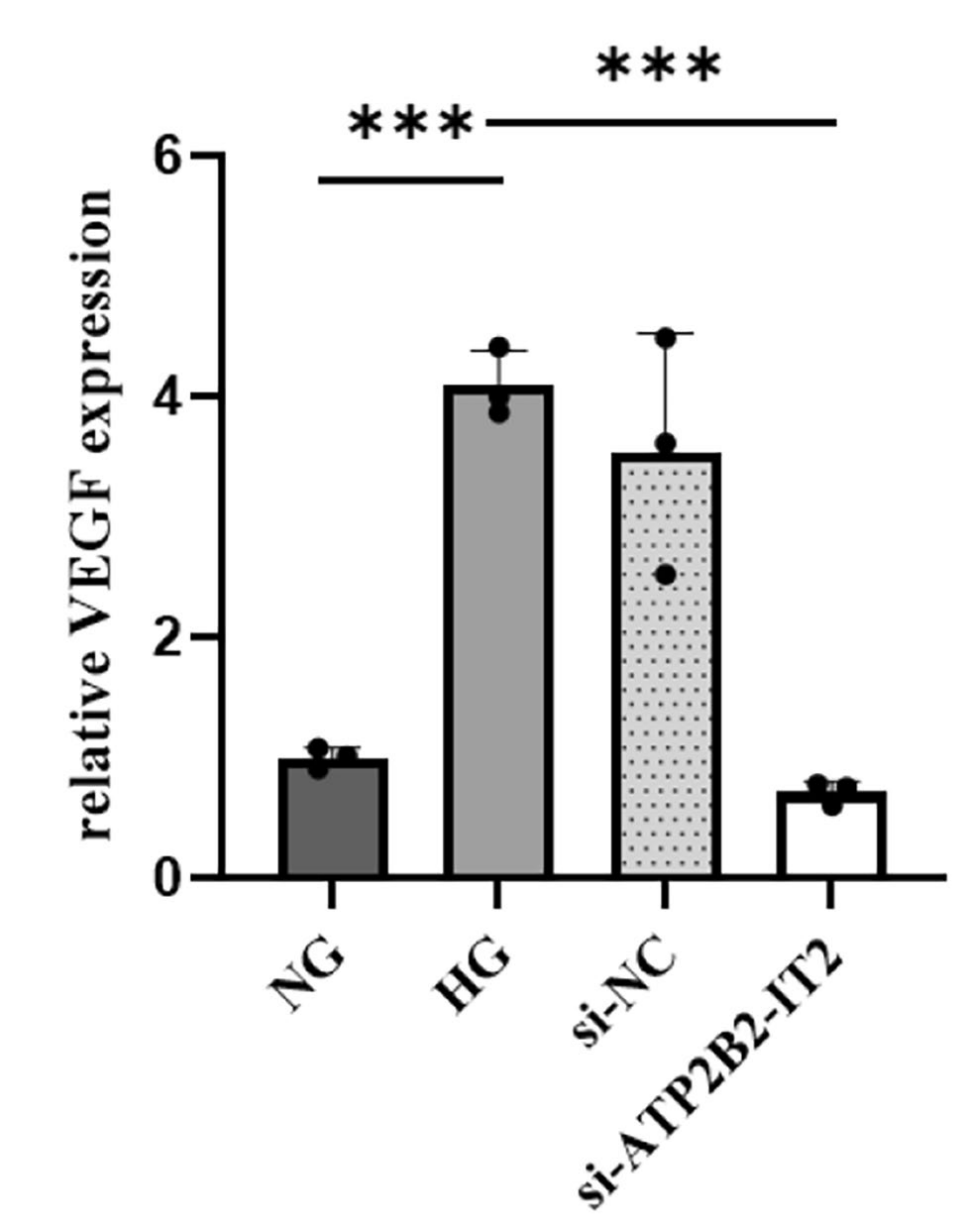
Expression of VEGF in HRMECs under high-glucose conditions after silencing of the lncRNA ATP2B2-IT2 (*n* = 3; *P < 0.05, **P < 0.01, ***P < 0.001)

According to the RT‒PCR results, VEGF expression was significantly lower in the HG + si-lncRNA ATP2B2-IT2 group than in the HG group (*P* < 0.05), while VEGF expression was not significantly different between the HG + si-lncRNA ATP2B2-IT2 group and the si-NC group (*P* > 0.05), as shown in Fig. 6.

## Discussion

Diabetes is a metabolic disease that poses a serious threat to human health, and this disease is characterized by high blood glucose [13, 14]. According to the prediction by the International Diabetes Federation, the number of people with diabetes will reach 537 million by 2021 and 783 million by 2045 [15]. With the increase in the number of people with diabetes, the incidence of DR has been increasing, so the poor vision of people with diabetes is a problem that requires prompt solutions.

LncRNAs, including those involved in ocular diseases, have become a hot topic in recent years. Wan et al. [16] reported that lncRNA-H19 can promote PDCD4 expression by sponging miR-21 and establish a competing endogenous RNA network in the ischemic cascade, ultimately initiating microglial pyroptosis and neuronal death in retinal ischemia/reperfusion injuries. According to Radhakrishnan R and Kowluru RA [17], the lncRNA MALAT1 can adjust the antioxidant defense in diabetic retinopathy through the Keap1/Nrf2 pathway, and inhibiting the lncRNA MALAT1 may help to protect the retina from oxidative damage and prevent or slow diabetic retinopathy. In previous work [18], vitreous humor (VH) specimens from patients who had idiopathic macular lentigines and who received vitrectomy were used as the control group, and VH specimens from PDR patients who underwent vitrectomy only and did not receive intravitreal injections of anti-VEGF before vitrectomy were used as the experimental group to assess the expression of lncRNAs by gene microarray. According to the gene microarray results, the two groups had significantly different expression of lncRNAs. Therefore, we speculated that lncRNAs may contribute to DR-related neovascularization, which is consistent with the findings of most existing studies. Based on previous work, we further screened genes with a fold change ≥ 1.5 and a *P* value < 0.05 in the microarray data and used RT‒PCR to determine the genes with the greatest differences in expression in HRMECs under normal and high-glucose conditions and found that the expression of the lncRNA ATP2B2-IT2 was greater under high-glucose conditions than under normal-glucose conditions. This finding was consistent with the results of the gene microarray in our early-stage research. According to a literature review, no studies have investigated the role of the lncRNA ATP2B2-IT2 in diabetic retinopathy. Therefore, the lncRNA ATP2B2-IT2 was selected for subsequent experiments. The results showed that the lncRNA ATP2B2-IT2 may contribute to the proliferation, migration, and angiogenesis of HRMECs under high-glucose conditions, and the si-lncRNA ATP2B2-IT2B2-IT2 may inhibit the proliferation, migration, and angiogenesis of HRMECs under high-glucose conditions.

VEGF is believed to be a major factor for treating PDR neovascularization [19, 20]. Some studies have shown that the aberrant production and release of VEGF induces and promotes the neovascularization process [21, 22]. VEGF-A, a subtype of VEGF, plays a critical role in this process [23, 24]. Therefore, VEGF-A was investigated in this study and was found to be increased in the HG group. This finding is consistent with the results of existing studies and indirectly proves the success of the cell studies in this research. After silencing of ATP2B2-IT2, the expression of VEGF under high-glucose conditions was significantly downregulated. Therefore, we argue that the ATP2B2-IT2 induces neovascularization through VEGF.

However, this research has several limitations. The effect of the ATP2B2-IT2 on neovascularization was proven only in cellular experiments, and no animal experiments were conducted to verify the effect. In future research, the effect of the ATP2B2-IT2 on neovascularization in animals should be discussed to explore the specific mechanism of neovascularization.

In conclusion, the lncRNA ATP2B2-IT2 is crucial in diabetic retinopathy from the early stage to the neovascularization stage of PDR, as it may induce retinal neovascularization by promoting VEGF. Currently, there is no effective early treatment for DR, and the lncRNA ATP2B2-IT2 may provide new potential targets and therapeutic ideas for the early treatment of DR to prevent its progression.

The regulatory role of lncRNAs provides a potential avenue for identifying novel therapeutic targets for diabetic retinopathy. For instance, lncRNAs such as MALAT1 and MIAT have been extensively studied and demonstrated to be involved in the pathogenesis of diabetic retinopathy [25]. However, the specific mechanisms by which lncRNAs exert their effects remain to be fully elucidated. The functions and potential downstream targets of lncRNAs selected as therapeutic targets have not been thoroughly investigated. Therefore, the clinical use of lncRNAs as targeted drugs still faces many challenges, and further research is needed to establish their efficacy in future studies.

### Electronic supplementary material

Supplementary Material 1

## Data Availability

All data generated or analysed during this study are included in this published article.
