# Supplementary figures and images for: Preliminary research on LncRNA ATP2B2-IT2 in neovascularization of diabetic retinopathy
Source: BMC Ophthalmol. 2024 Jun 21;24:267. doi: 10.1186/s12886-024-03523-5 (PMC11191339; doi:10.1186/s12886-024-03523-5)

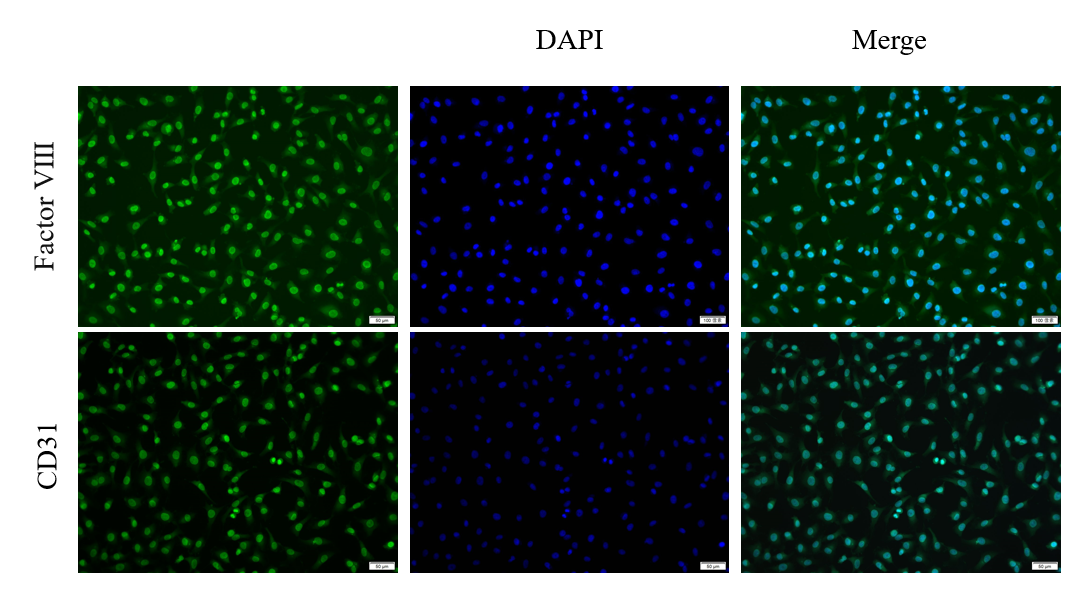

Supplement: Supplementary file 1 — Supplementary Material 1 [file 12886_2024_3523_MOESM1_ESM.png]
